# Supplementary figures and images for: Phylogeny and Historical Biogeography of the East Asian Clematis Group, Sect. Tubulosae, Inferred from Phylogenomic Data
Source: Int J Mol Sci. 2023 Feb 3;24(3):3056. doi: 10.3390/ijms24033056 (PMC9917980; doi:10.3390/ijms24033056)

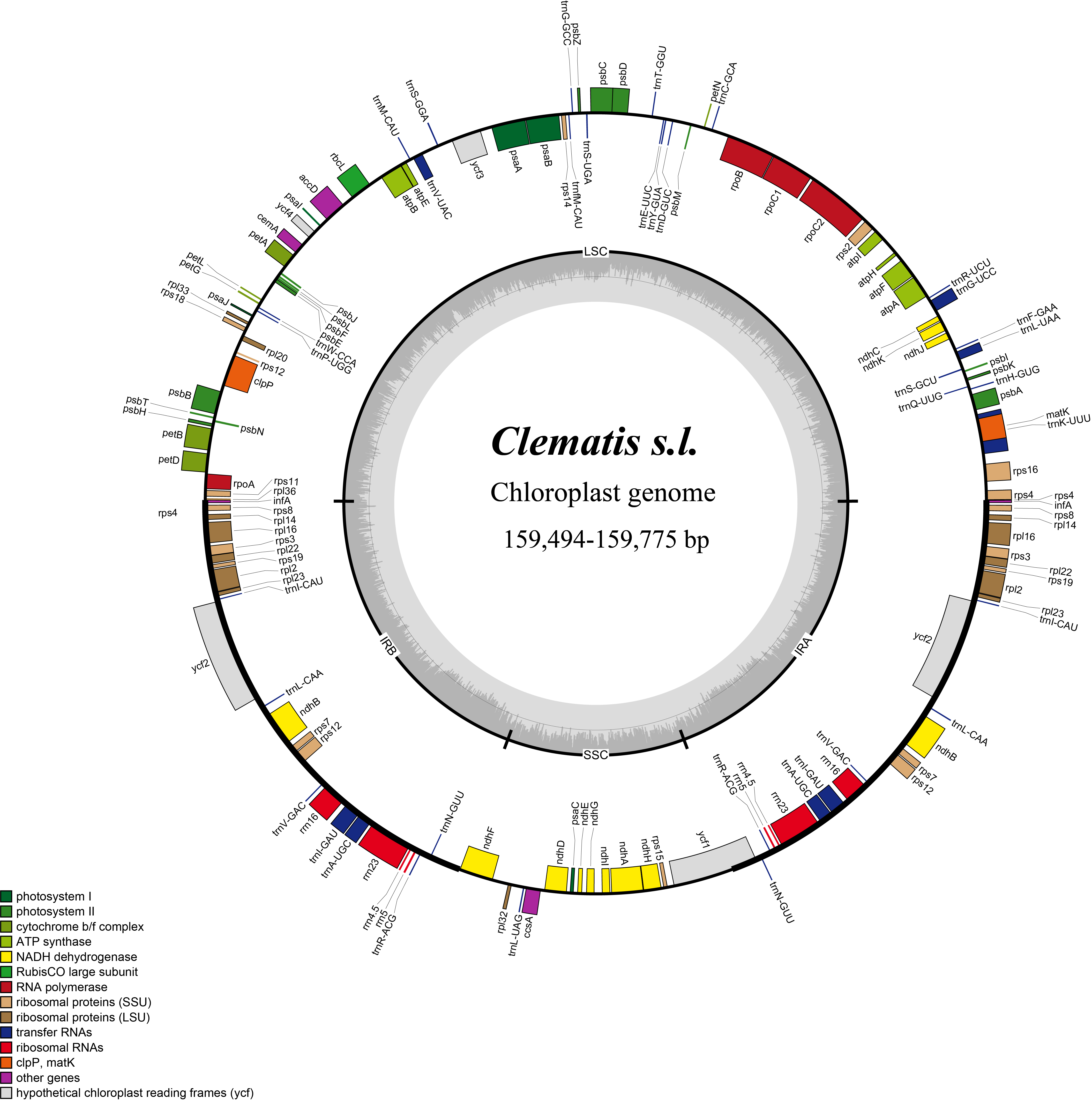

Supplement: Supplementary file 1 [file ijms-24-03056-s001.zip › Supplementary Files/Figure S1 Plastid genome map.jpg]

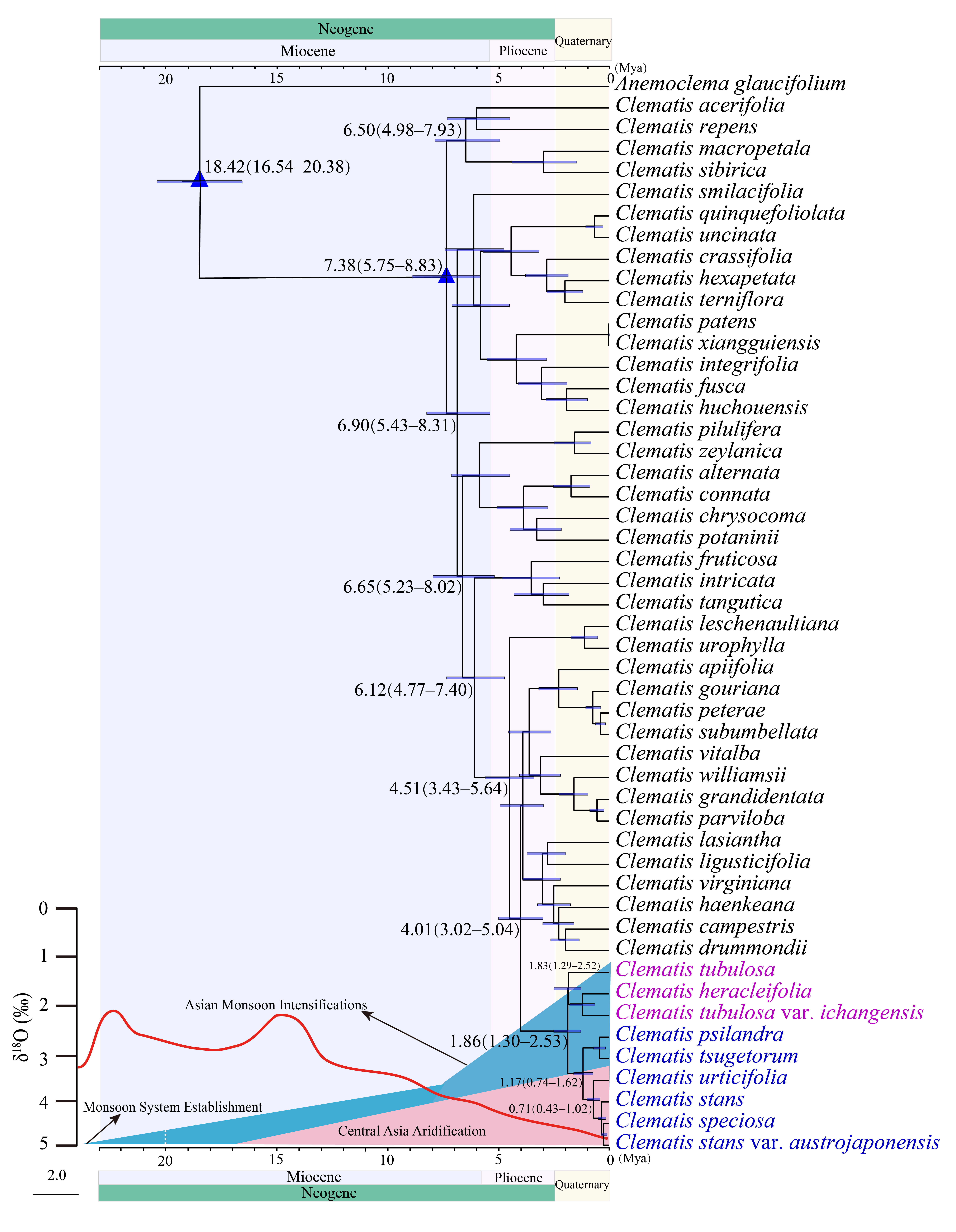

Supplement: Supplementary file 1 [file ijms-24-03056-s001.zip › Supplementary Files/Figure S2 beast_cp50_0131.jpg]
